# Preservation of Atlantic Salmon (*Salmo salar*) Fillets Using Jasmine Essential Oil-Loaded Nanoemulsions Stabilized with a Whey Protein/Oxidized Corn Starch Complex

**DOI:** 10.3390/foods14173024

**Published:** 2025-08-28

**Authors:** Jie Shen, Song Li, Peng Shi, Yibin Zheng, Jun Mei

**Affiliations:** 1 College of Food Science and Technology, Shanghai Ocean University, Shanghai 201306, China; 2234105@st.shou.edu.cn (J.S.);; 2 National Experimental Teaching Demonstration Center for Food Science and Engineering, Shanghai Ocean University, Shanghai 201306, China; 3 Shanghai Engineering Research Center of Aquatic Product Processing and Preservation, Shanghai 201306, China; 4 Shanghai Professional Technology Service Platform on Cold Chain Equipment Performance and Energy Saving Evaluation, Shanghai 201306, China

**Keywords:** jasmine essential oil, nanoemulsion, oxidized corn starch, whey protein, Atlantic salmon (*Salmo salar*), refrigerated preservation, quality enhancement

## Abstract

This study evaluated the ability of a jasmine essential oil (JEO)-loaded nanoemulsion alone (WOM) and combined with modified atmosphere packaging (WOM-MAP) to extend the shelf life and maintain the quality of Atlantic salmon (*Salmo salar*) at 4 °C. The WOM was stabilized with oxidized corn starch (OCS) and whey protein (WP), and had a particle size of 255.7 nm and zeta potential of −25.43 mV. The treated salmon fillets were periodically analyzed for spoilage extent, texture, protein structure, and other quality parameters. The findings of this study showed that the salmon fillets treated with the WOM, particularly when combined with the MAP, was able to retain their original texture and taste to a great extent. Compared to the control check group (CK), the WOM and WOM-MAP treatments reduced bacterial counts by 2.1 log CFU/g and 2.4 log CFU/g, respectively, by the 20th day. Both the WOM and WOM-MAP treatments exhibited lower total volatile basic nitrogen and K values, prevented protein structural changes, and reduced free amino acid breakdown. These results indicate that the WOM effectively extends the shelf life and maintains the quality of Atlantic salmon, offering a promising natural preservative to preserve the quality and safety of seafood.

## 1. Introduction

Atlantic salmon (*Salmo salar*) holds significant commercial importance due to its appealing flavor profile and rich ω-3 fatty acid content [1], making it a popular choice among consumers around the world. However, salmon is susceptible to microbial contamination—notably by *Pseudomonas fluorescens* (*P*. *fluorescens*)*,* which is the organism that specifically causes spoilage in refrigerated salmon [2,3]—as well as oxidative degradation [4] during storage, processing, and transportation. These quality deterioration processes not only reduce the product value but also pose potential food safety risks [5]. While physical preservation techniques can fulfill the demands of large-scale commercial storage, they have an inherent disadvantage: freezing results in the formation of ice crystals, leading to mechanical damage and the loss of texture [6,7]. On the other hand, when using chemical preservatives, attention needs to be paid to the dosage, as excessive amounts can pose health risks [8]. Therefore, exploring effective and natural fresh preservation techniques is essential to extend the shelf life of salmon and maximize its commercial potential.

Current advances in food preservation technology have focused on developing natural antibacterial agents to improve product safety and quality while extending shelf life [9]. Jasmine (*Jasminum* spp.), a widely appreciated flowering plant known for its characteristic floral aroma, contains bioactive compounds in its essential oil (JEO), particularly α-pinene, linalool, and cinnamaldehyde, and has well-documented antibacterial, antifungal, and antioxidant properties [10,11]. However, JEO has several limitations when used in its free form, particularly due to its high volatility [12]. Furthermore, environmental stressors such as heat, pH, and oxygen exposure can compromise the stability of its bioactive compounds, leading to degradation of its antioxidant and antimicrobial activities [13]. Encapsulation technology offers a promising solution to overcome these challenges and enhance JEO’s functional efficacy [14].

Creating a nanoemulsion (NE) is an effective way for encapsulating unstable active ingredients such as essential oils [15]. Due to their unique properties including high stability, a small droplet size, and large surface area [12], NEs enable controlled release and enhance the bioavailability of lipophilic bioactive compounds [13,16]. A variety of substances can serve as carrier materials in NE formulations. In the past, inorganic particles and synthetic polymer nanoparticles were primarily used to stabilize NEs but resulted in NEs with poor biocompatibility that could not be degraded [17]. Exploring carbohydrates, lipids, protein, gums, and food-grade polymers [18,19] to stabilize NEs and expanding their applications have become major research focuses.

Whey protein (WP) possesses a unique structural configuration with a core hydrophobic domain [20]. This structure enables WP to effectively bind both hydrophobic (e.g., essential oils and spices) and weakly polar small molecules (e.g., fat-soluble vitamins, fatty acids, and functional peptides). Oxidized corn starch (OCS), which is synthesized from corn starch and oxidants, is widely utilized in the food, biotechnology and pharmaceutical industries due to its favorable physicochemical properties, including high yields, low viscosity, high clarity, and low-temperature stability [21]. Compared with single particle stabilization, OCS-WP complexes synergistically combine the advantages of both components while mitigating their individual limitations: they not only reduce the structural dissociation of proteins at the adsorption interface but also decrease the hydrophilicity of polysaccharides [22,23]. The OCS and WP form a stable complex that can be employed to prepare a jasmine essential oil-loaded nanoemulsion (JEO-NE) to enhance the JEO’s biological activity [12].

Essential oil-based NEs are widely used, and their antioxidative and antibacterial properties have been shown to be effective in preserving salmon. There is a paucity of studies that encapsulated JEO using NE technology. The objective of this study is to explore the potential application of JEO-NEs as a biological preservative for salmon and evaluated the physicochemical quality and microbiological changes in the salmon.

## 2. Materials and Methods

### 2.1. Preparation of Jasmine Essential Oil-Loaded Nanoemulsions

#### 2.1.1. Synthesis of WP-OCS Mixture

A 4% (*w*/*v*) aqueous whey protein solution was prepared by dissolving bovine whey (Macklin Biochemical Co., Ltd., Shanghai, China) in deionized water. Oxidized corn starch (Henan Hengrui starch Technology Co., Ltd., Henan, China) was dissolved in deionized water to prepare an aqueous 1% (*w*/*v*) oxidized corn starch solution. The two solutions were combined at a volume ratio of 1:4 to create a WP-OCS mixture (WO). The WP-OCS mixture was continuously stirred with a magnetic stirrer for 24 h at room temperature. After adjusting the pH of the WP-OCS mixture to 7, it was stored in a refrigerator (BD/BC-568DKEM, He Fei Midea Refrigerator Co., Ltd., Hefei, China) at 4 °C until further use.

#### 2.1.2. Nanoemulsion Preparation

**Figure 1:**
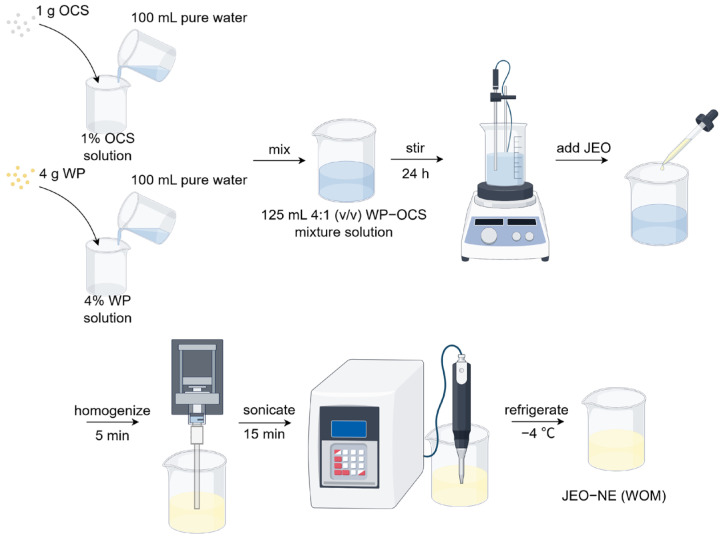
Flowchart of JEO-loaded nanoemulsion preparation.

After adding different volumes of jasmine essential oil (purity: 100%; Ji’an Huashuo Fragrance Oil Co., Ltd., Ji’an, China) to the WP-OCS mixture to form 0.5%, 1%, 2%, 4%, and 8% JEO crude emulsions (*v*/*v*), the crude emulsions were homogenized for 5 min at 10,000 rpm in an ice bath using a high-speed homogenizer (T 25 digital ULTRA-TURRAX, IKA, Staufen, Germany). Finally, the crude emulsions were sonicated using an ultrasonic cell breaker (power: 150 W; time: 15 min; treatment protocol: on for 2 s and off for 2 s) to obtain jasmine essential oil-loaded nanoemulsions stabilized with a whey protein/oxidized corn starch complex (WOMs). The emulsions were stored in a refrigerator (BD/BC-568DKEM, He Fei Midea Refrigerator Co., Ltd., Hefei, China) at 4 °C until further use. The preparation process is shown in Figure 1.

### 2.2. Characterization of Nanoemulsions

The particle size, polydispersity index (PDI), and ζ potential of the nanoemulsions were determined using a Malvern laser particle sizer (Zetasizer pro, Malvern Instruments, Malvern, UK) and the method described by Sun et al. [24].

### 2.3. Analysis of Antibacterial Activity

#### 2.3.1. Preparation of Bacterial Suspensions

*Staphylococcus aureus (S. aureus), Shewanella putrefaciens* (*S*. *putrefaciens*), and *P. fluorescens* were evenly smeared on LB agar (Qingdao Hi-Tech Industrial Park Hope Bio-Technology Co., Ltd., Qingdao, China) plates and cultivated at 37 °C overnight. Then, a single bacterial colony was transferred into 10 mL of LB broth (Qingdao Hi-Tech Industrial Park Hope Bio-Technology Co., Ltd., Qingdao, China) and cultivated at 37 °C for another 16 h. During the cultivation process, the OD of the bacterial suspension was measured until it reached 0.5, which is equivalent to a concentration of 10^8^ CFU/mL.

#### 2.3.2. Determination of Inhibition Zone

Inhibition zone determination was performed according the protocol of Ma et al. [25]. A 0.1 mL volume of each of the three bacterial suspensions was added and spread on different Mueller–Hinton agar plates (Qingdao Hi-Tech Industrial Park Hope Bio-Technology Co., Ltd., Qingdao, China). A 100 μL volume of the JEO-NE was added into sterile Oxford cups and incubated at 37 °C. After 24 h, the diameters of the inhibition zones were measured.

#### 2.3.3. Determination of Minimum Inhibitory Concentration (MIC)

The minimum inhibitory concentrations (MICs) of the jasmine essential oil nanoemulsions were measured using the protocol of Li et al. [26]. Different concentrations (20, 10, 5, 2.5, 1.25, 0.625, and 0.3125 μL/mL) of the nanoemulsions were mixed with 100 μL LB medium and then 100 μL of a bacterial suspension (10^6^ CFU/mL) was added. The solution was transferred into 96-well plates. After cultivating at 37 °C for 24 h, the OD_600_ was measured using an enzyme-linked spectrophotometer (SPARK, Tecan, Männedorf, Switzerland). The controls, which including gentamicin as the positive control, the LB medium without bacteria as a sterile negative control, the LB medium with the relevant bacteria as the growth negative control, and the WO as the emulsifier control, were treated using the same steps and the OD600 was measured. The MIC of each bacterium was determined in triplicate. The MIC is defined as the lowest concentration at which the resulting OD value matches the initial OD value (ΔOD_600_ ≤ 0.5).

### 2.4. Preparation of Fillet

The fresh salmon (6 kg) was provided by Shanghai Chengyu Trading Co., Ltd., Shanghai, China, and was transported to the laboratory in an insulated box at 0–4 °C over a period of 30 min where it was cut into pieces (20 mm × 20 mm × 10 mm). Each part of the fillets was randomly divided into four groups (Table 1): Control Check (CK), WO, WOM, and MAP-WOM groups. The fish fillets in the WO group were immersed in the WP-OCS mixture for 30 min, and the WOM and MAP-WOM groups were immersed for the same amount of time in the JEO-loaded nanoemulsion. After processing, the CK, WO, and WOM groups were directly sealed and put in a 4 °C refrigerator. The MAP-WOM group was refrigerated after being placed in a modified air package (60% N_2_/40% CO_2_). The indicators were measured on days 0, 4, 8, 12, 16,and 20.

### 2.5. Sensory Evaluation

The sensory evaluation was conducted using the quality index technique (QIM). The evaluation was performed by 20 sensory experts who were professionally trained according to ISO standard No. 8586:2023, guaranteeing a consistent evaluation. The evaluation was focused on texture, color, odor, and overall acceptability. The experts scored these indicators on a 5-point scale with 5 indicating the best quality and 1 indicating the lowest quality. A score of 3 represented the minimum acceptability threshold [27]. Scores below 3 were deemed unacceptable.

### 2.6. Texture Profile Analysis (TPA)

Following the method of Dong et al. [28] with appropriate modifications, the texture of the salmon fillets (20 mm ×20 mm ×15 mm) was measured using a TA-XT plus texture analyzer (Stable Micro System Ltd., Godalming, UK) equipped with a 50 mm probe (P/50) at room temperature. The probe was compressed twice at a constant rate of 1 mm/s with an interval of 5 s to 50% of the original height, and the results for each part of salmon fillet were recorded separately.

### 2.7. pH

The method of Liu et al. [29] was used: 5 g of salmon was homogenized in 45 mL of distilled water. After filtration, each sample was analyzed in triplicate using a pH meter (FE22-Meter, Mettler Toledo, Columbus, OH, USA).

### 2.8. Total Volatile Basic Nitrogen (TVB-N) Content

The TVB-N content was determined according to the National Standard of China (GB 5009.228-2016) [30]. A 5 g fish sample was mixed with 0.5 g of magnesium oxide (MgO) and was analyzed in triplicate using a nitrogen analyzer (Kjeldahl 8400, FOSS, Hillerød, Denmark). The result was expressed as mg N/100 g.

### 2.9. Total Viable Counts (TVC)

The TVC in the fish was measured in accordance with the National Standard of China (GB 4789.20-2024) [31]. The TVC samples were cultured in plate count agar (Qingdao Hi-Tech Industrial Park Hope Bio-Technology Co., Ltd., Qingdao, China) plates at 30 °C for 48 h before counting. The result is presented as log CFU/g.

### 2.10. Color

Surface color, which is a salmon quality indicator, was analyzed using a colorimeter (YS6010, Shenzhen 3nh Technology Co., Ltd., Shenzhen, China). Luminance (L*), redness (a*), and yellowness (b*) values for each part of the salmon fillets were recorded every four days. Each sample was tested in parallel three times.

### 2.11. Free Amino Acid (FAAs) Content

The salmon samples were prepared according to the method of Cao et al. [32]. A 2 g fish sample was mixed with 10 mL of TCA (Sinopharm Chemical Reagent Co.), homogenized, and then centrifuged (10,000× *g*, 10 min). The centrifugation was repeated and the mixture was diluted to a final volume of 25 mL. The processed sample was filtered with a 0.22 μm filter and analyzed using an amino acid analyzer (LA8080 Ultra High-Speed Automatic Amino Acid Analyzer, Hitachi, Tokyo, Japan).

### 2.12. K Value

We used the test method of Yang et al. [33] for ATP-related compounds that uses high-performance liquid chromatography (HPLC) (Waters 2695, Milford, CT). A 5 g fish sample was mixed with 10 mL of 10% perchloric acid (PCA) and homogenized. The supernatant was taken after centrifugation (4 °C 8000× *g* 10 min) and 10 mL of 5% PCA was added to the precipitate, which was then centrifuged (4 °C 8000× *g* 10 min) three times. All three supernatants were combined, and 15 mL of ultra-pure water was added. The solution was diluted to a final volume of 50 mL after adjusting the pH to 6.5. The processed sample was filtered with a 0.22 μm filter and analyzed by HPLC. The formula for the K-value calculation was as follows:

$$K\;value\left(\%\right)=\frac{HxR+Hx}{ATP+ADP+AMP+IMP+HxR+Hx}\times 100\%$$

where Hx is the hypoxanthine content; HxR is the hypoxanthine ribonucleoside content; IMP is the inosine monophosphate content; AMP is the adenosine monophosphate content; ADP is the adenosine diphosphate content; and ATP is the adenosine triphosphate content.

### 2.13. Extraction of Myofibrillar Proteins (MPs)

Myofibrillar proteins were extracted using the method of Yu et al. [34]. A 2 g sample of minced salmon was added to 20 mL of pre-cooled buffer (0.05 mol/L Tris-HCl, pH 7.6), homogenized, and then centrifuged at 10,000× *g* for 15 min at 4 °C to obtain a precipitate. The centrifugation was repeated once following the steps above. The obtained precipitate was added to pre-cooled buffer (0.05 mol/L Tris-HCl and 0.6 mol/L KCl, pH 7.6), homogenized, extracted at 4 °C for 3 h, and centrifuged under the same conditions as above and the supernatants were collected.

#### 2.13.1. MP Carbonyl Group Content

The carbonyl group content was determined according to the method of Xu et al. [35], which uses 2,4-dinitrophenylhydrazine derivatization. The results are given in μmol/g.

#### 2.13.2. MP Sulfhydryl Group Content 

The assay method of Shi et al. [36] was used to measure the MP sulfhydryl group content, which was detected using the 5,5-dithiobis-2-nitrobenonic acid (DTNB) chromogenic reaction. The results are given in μmol/g.

#### 2.13.3. Ca^2+^-ATPase Activity of MPs

Ca^2+^-ATPase was measured using a micro-assay kit from the Nanjing Jiancheng Bioengineering Institute (Nanjing, China). The results are given in μmol Pi/mg prot/hour.

#### 2.13.4. MP Secondary Structures

The method of Shi et al. [37] was used. The processed samples were analyzed by circular dichroism spectroscopy (Chirascan plus, Applied Photophysics, Leatherhead, UK) in the 160 nm–280 nm wavelength range.

#### 2.13.5. MP Tertiary Structures

The method of Ma et al. [38] using fluorescence spectroscopy (F-7100, Hitachi, Tokyo, Japan) was used to analyze the MP tertiary structures (excitation: 295 nm; emission: 300–400 nm; slit width: 5 nm; scan speed: 1200 nm/min).

### 2.14. Statistical Analysis

Each experiment was carried out in triplicate. The results are shown as the mean ± standard deviation. IBM SPSS Statistics 27 software was used for the statistical analysis of the data, and significant differences (*p* < 0.05) were determined using one-way ANOVA and Duncan’s multiple range test. Origin 2023 software was used to generate the line graphs.

## 3. Results

### 3.1. Properties of Jasmine Essential Oil-Loaded Nanoemulsions

#### 3.1.1. Appearance, Particle Size, PDI, and ζ Potential

**Figure 2:**
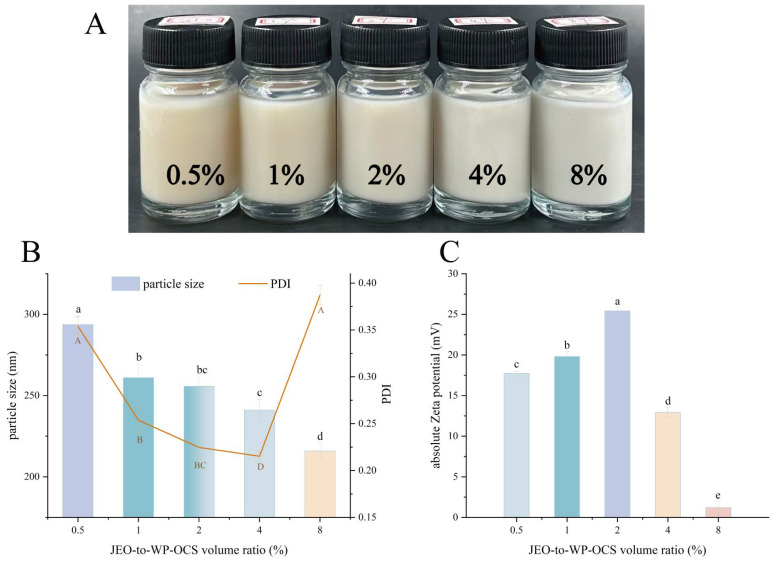
The appearance of the newly prepared JEO-NEs (**A**) with different concentrations (from left to right: 0.5%, 1%, 2%, 4%, and 8% (*v*/*v*)) and the effects of different JEO-to-WP–OCS ratios on the particle size (**B**) and zeta potential (**C**) of the JEO nanoemulsions. Values with different letters and different colors are significantly different (*p* < 0.05).

Figure 2 shows a picture of the JEO-loaded nanoemulsions with different JEO ratios. The newly prepared JEO nanoemulsions were mainly white. As the JEO ratio decreased, the color gradually changed from white to light yellow.

The particle size of an NE is a critical quality parameter. Nano-sized NEs possess a large surface area, which enhances their antibacterial and antioxidative activities [39]. The various formulations exhibited particle sizes (Figure 2A) within the nanoscale range of 200–300 nm. Figure 2A illustrates the effect of the JEO-to-WP–OCS ratio (*v*/*v*) on the particle size. As the proportion of the JEO increased, the particle size decreased. The smallest particle size was 215.9 nm with a PDI of 0.4871 (8% JEO). The ζ potential reflects the surface charge density [40] and serves as an indicator of nanoemulsion stability [41]. A higher absolute ζ potential enhances stability by preventing particle aggregation through electrostatic repulsion [42,43]. Although the 8% JEO formulation had the smallest particle size (215.9 nm), its ζ potential was close to 0 mV, indicating severe instability [44] (Figure 2A,B). In contrast, the formulation with the third-smallest particle size (2% JEO; particle size of 255.7 nm and PDI of 0.2247) exhibited the highest ζ potential (−25.43 mV). Its PDI of 0.2247 indicates minimal aggregation and flocculation, further confirming its stability [44]. In summary, the optimal JEO-to-WP–OCS ratio was 2%, which strikes a balance between particle size and stability. Deviating the ratio from 2% inevitably led to instability. A higher ratio resulted in droplet aggregation and a lower ratio resulted in a larger particle size [25,45].

#### 3.1.2. Determination of Inhibitory Activity

According to previous experiments, JEO exhibits strong antibacterial activity. Loading the JEO into an NE further enhanced its antibacterial efficacy. Antibacterial experiments were conducted against *S. aureus*, *S. putrefaciens*, and *P. fluorescens*. *P. fluorescens* is a spoilage organism specific for refrigerated salmon [2,3] and *S. putrefaciens* is involved in the spoilage process of various marine fish, especially those preserved via refrigeration [46]. Both *P. fluorescens* and *S. putrefaciens* are Gram-negative bacteria [47,48] and *S. aureus* is a Gram-positive bacteria.

All the tested bacterial strains displayed clear inhibition zones (Table 2), with the largest zones observed for *Pse. fluorescens*. The JEO-loaded nanoemulsion had an MIC of 10 μL/mL, confirming its potent antibacterial activity. According to the MIC results, the WO group did not demonstrate any antibacterial activity against the three bacteria strains, further proving that the antibacterial activity was mainly from the bioactive compounds in the JEO [11]. Moreover, the JEO- loaded nanoemulsion also exhibited potent antibacterial activity against *S. aureus* and *S. putrefaciens*, with MICs of 5 μL/mL and 10 μL/mL. Due to the difference between Gram-negative and Gram-positive bacteria in the structure of their cell walls [49], the JEO-loaded NE might target specific components of the cell wall of Gram-positive bacteria, leading to a stronger inhibition of *S. aureus*. Consequently, the JEO-loaded NE had an inhibitory effect on both Gram-positive and Gram-negative bacteria, indicating broad-spectrum antibacterial activity.

### 3.2. Sensory Evaluation

**Figure 3:**
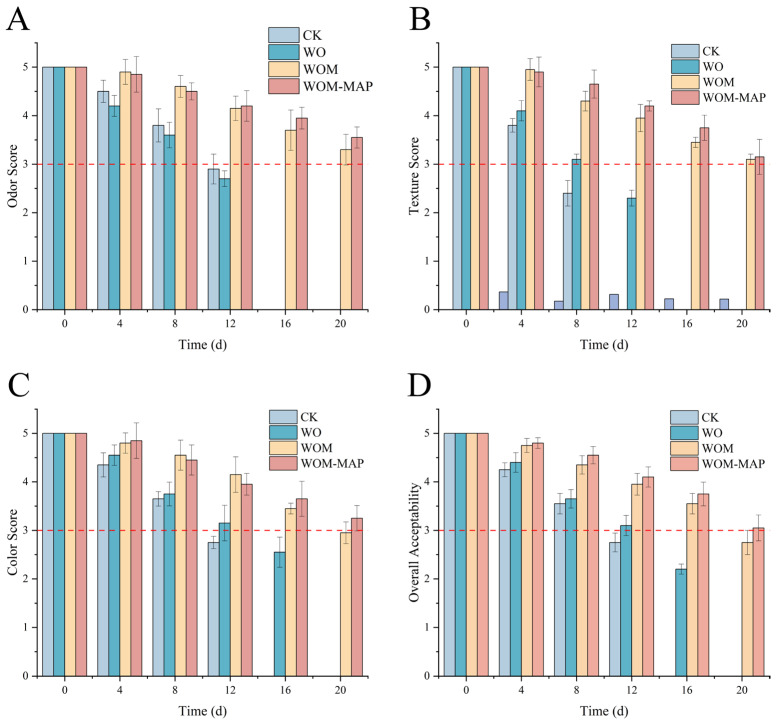
Changes in sensory qualities ((**A**) odor, (**B**) texture, (**C**) color, (**D**) overall acceptability) using different packaging methods during storage.

The sensory qualities of the salmon samples stored using different packaging methods are shown in Figure 3. The sensory score significantly decreased in all groups during storage, which is consistent with the results of the study by Zhang et al. [1]. The CK group reached a score of 3 on the 8th day, which is considered unacceptable, and was below the scores of the WOM and WOM-MAP groups throughout the 20-day storage period. The sensory evaluation of the WOM-MAP group was the best and did not decline to an unacceptable level even on the 20th day. During storage, the growth of microorganisms caused the decline in the sensory attributes (texture, odor, and color) [36]. The deterioration in color was mainly because of lipid and protein oxidation, resulting in a dark color [1,50]. Additionally, these oxidation products (e.g., peroxides and ketones) also led to an undesirable flavor and the protein denaturation resulted in a loose structure and poor texture. Consequently, the JEO-loaded nanoemulsion effectively inhibited microbial growth and slowed down the degradation of protein and the accumulation of volatile substances [38]. In addition, the unique fragrance of JEO added a pleasant smell, which increased the acceptance. The WOM and WOM-MAP effectively slowed down the texture deterioration and color loss, and prevented the release of bad odors, significantly enhancing the overall acceptability to consumers.

### 3.3. Texture Profile Analysis (TPA)

**Figure 4:**
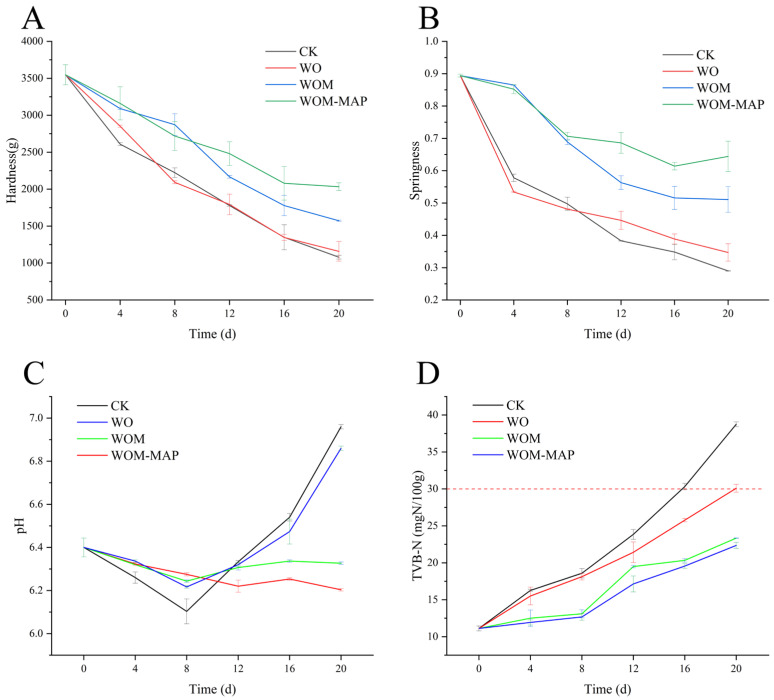
Texture profile analysis (TPA): hardness (**A**), springiness (**B**), pH (**C**), and TVB-N (total volatile basic nitrogen) content (**D**) using different packaging methods during storage.

The TPA parameters are important indicators of fish texture. Figure 4A,B displays the TPA results for the four experimental groups at different days during storage. Hardness and springiness showed similar trends in all the groups: they progressively decreased during storage. This is consistent with the findings of the study by Huang et al. [51]. On day 0, the hardness and springiness values were 3714 g and 0.894 mm, respectively. Protein structure plays a crucial role in fish texture, and during storage, protein degradation occurs, leading to denaturation, structural loosening, and a loss of texture [52]. On day 20, the hardness and springiness values were significantly lower in the WOM (1572.613 g, 0.51 mm) and WOM-MAP (2732.613 g, 0.64 mm) groups compared to the CK (1078.68 g, 0.29 mm) and WO (1158.259 g, 0.35 mm) groups. The reproduction of microorganisms is the other main factor in myofibril destruction and quality deterioration [53]. This demonstrated that the JEO-loaded NE exhibited an inhibitory effect on protein degradation and denaturation. Furthermore, the data indicated that the JEO-loaded NE slowed the decline in springiness more effectively than that of hardness. This difference may be attributed to the interaction between proteins and their surrounding hydrated layer, which contributes to springiness [54,55]. Additionally, the packaging materials may interfere with this interaction, which influenced the observed trends. The WOM evidently slowed down the loss of hardness and springiness, and the fish retained its original texture due to the WOM’s antibacterial activity and ability to maintain protein structures.

### 3.4. pH

pH is a reliable indicator of preservation quality in aquatic products. The initial pH value of the salmon samples was 6.4. As shown in Figure 4C, the pH values of all the groups showed the same trend, initially decreasing and then increasing, though at different rates. This result is consistent with the report by Zhang et al. [1]. There were no significant differences in the initial pH decline among the groups. The lowest value (6.10) was observed in the CK group on the 8th day. This decrease could be attributed to lactic acid accumulation and dissolved CO_2_ due to microbial fermentation [56]. In addition, the inorganic phosphate from ATP degradation also contributed to the decrease in pH [57]. In contrast, the subsequent pH increase was likely driven by the degradation of amino acids and the production of volatile nitrogenous compounds, including biogenic amines, ammonia, dimethylamines, and trimethylamines [58]. During the rising pH stage, the WOM and MAP-WOM groups exhibited a slower pH increase than the untreated groups (CK and WO). On the 20th day, the pH values reached 6.96 (CK) and 6.86 (WO), whereas the WOM and MAP-WOM groups registered notably lower values (6.33 and 6.20, respectively). In the treated groups, the degradation of amino acids by microbes was effectively inhibited, leading to the production of bacterial proteases (e.g., *P. fluorescens* P15 protease) and decreased release of volatile nitrogenous compounds [57,59]. Therefore, the WOM and WOM-MAP groups showed smaller variations in pH compared to CK. This effect may be linked to the bioactive compounds in the JEO, which likely disrupted microbial membrane integrity. Due to their hydrophobicity, essential oils can enter into bacterial cell membranes, increasing permeability, inducing the leakage of cellular contents, and ultimately causing cell death [60]. As a result, the WOM treatment was better at maintaining the quality of the salmon during storage.

### 3.5. Total Volatile Basic Nitrogen (TVB-N) Content

The TVB-N content refers to the content of nitrogenous compounds, including ammonia, dimethylamine, and trimethylamine, that contribute to spoilage [61,62]. Its value serves as a key indicator of protein and amine degradation caused by microbial or enzymatic activity [3,63]. The upper acceptable limit for marine fish is 30 mg N/100 g. As shown in Figure 4D, the initial TVB-N value was 11.12 mg N/100 g. During storage, the TVB-N values gradually increased. The CK and WO groups exceeded the upper limit on days 12 and 20, respectively, whereas the WOM and WOM-MAP groups remained below the threshold throughout the 20-day period. This rise in TVB-N content was attributed to bacterial/enzymatic deamination and decarboxylation of proteins, releasing alkaline nitrogenous compounds [53]. The suppressed TVB-N accumulation in the JEO-treated groups (WOM and WOM-MAP) demonstrates that JEO could inhibit protein degradation. This effect likely stems from the antibacterial and antioxidative activities of JEO [63], which reduce oxidative deamination by microbes, thereby limiting TVB-N formation [64]. Notably, all the groups showed minimal TVB-N increases during the first 8 days, coinciding with the pH decline and release of proteases, which explains the later surge in pH values [65], a trend that was corroborated by our experiment results. Chen et al. [66] evaluated the impact of a curcumin-mediated photodynamic treatment on salmon preserved by chilling. The findings indicated that the treatment resulted in a 4-day extension of the salmon’s shelf life, consistent with the WOM treatment in this study. This further confirms that the WOM effectively inhibited the degradation of amino acids due to the JEO’s strong antibacterial and preservative activities.

### 3.6. Total Viable Counts (TVC)

**Figure 5:**
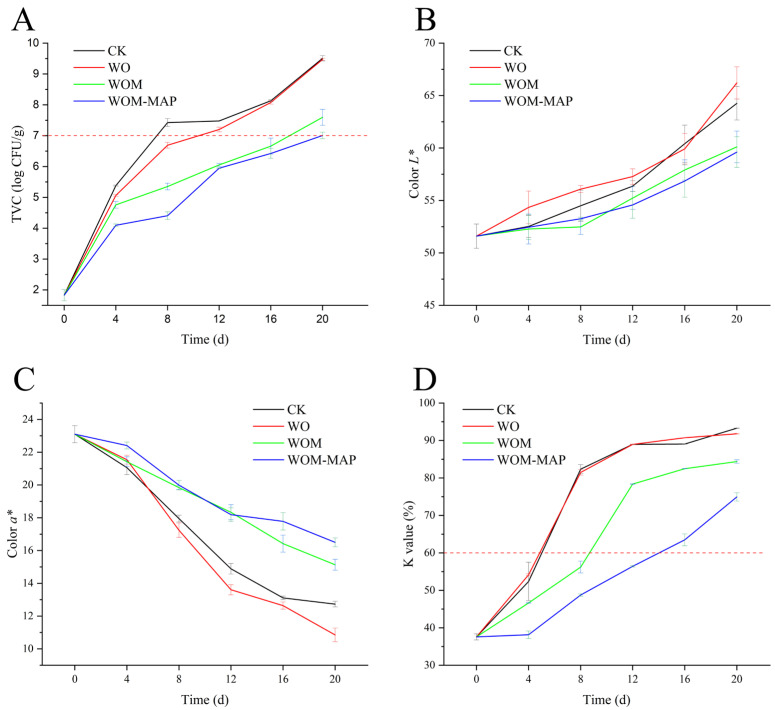
Changes in TVC (total viable count) (**A**), color L* (**B**), color a* (**C**), and K values (**D**) using different packaging methods during storage.

The TVC changes in the salmon during storage are presented in Figure 5A. The initial TVC was 1.83 log CFU/g. According to the International Commission on Microbiological Practices in Food (ICMSF), the safety limit for fresh fish is 7 log CFU/g [67]. The TVCs of the salmon samples increased at varying rates during storage. After the 4th day, the TVC growth rate was significantly slower in the JEO-treated groups compared to the untreated groups. The CK and WO groups exceeded the limit on the 8th and 12th day, respectively, while the WOM group exceeded the limit on the 20th day and the MAP-WOM group remained below the threshold even on the 20th day. These results suggest that the starch/protein complex nanoemulsion may have allowed for the sustained release of the JEO, thereby extending its antibacterial effects during the salmon storage. This finding aligns with those of a past study by Liu et al. [68], which demonstrated the prolonged antimicrobial activity of essential oil nanoemulsions. Zhang et al. [1] explored the application potential of a chitosan (CS)-based nanoemulsion combined with melleolides in fresh salmon preservation, extending the shelf life from 6 days to 12 days. The JEO-loaded nanoemulsion effectively extended the shelf life from 8 days to 20 days, indicating potent antibacterial activity.

### 3.7. Color

Fish color serves as the primary determinant of visual appeal. The color attributes redness (a*) and yellowness (b*) are associated with the myoglobin pigment content, while lightness (L*) relates to muscle tissue structure [69]. As shown in Figure 5B, the L* value exhibited a continuous increase throughout storage. During salmon storage, the surface color typically darkens due to protein denaturation and microbial melanin secretion [66]. However, protein hydrolysis induces structural changes that enhance light scattering, leading to increased L* values [70]. The impact of protein hydrolysis appears to be more significant than melanin release, ultimately resulting in an increase in L* values. The WOM and WOM-MAP groups demonstrated a slower L* value increase compared to the CK group. This suggests that the WOM treatment effectively slowed protein hydrolysis, likely through microbial inhibition and structural preservation mechanisms.

The a* value exhibited an initial decrease followed by a subsequent increase during storage (Figure 5C). These changes were significantly correlated with the metmyoglobin content [71]. The Mb redox state primarily determines the a* value due to the three forms of Mb: deoxymyoglobin (DeoMb, purple), oxymyoglobin (OxyMb, bright red), and metmyoglobin (MetMb, dark brown) [70]; their relative proportions governing the color of the fish [72]. Additionally, the red color of salmon is also due to the pigment astaxanthin [73,74]. The observed a* value reduction resulted from oxidative processes affecting both myoglobin and astaxanthin levels. The WOM and WOM-MAP treatments significantly attenuated this decline compared to the CK and WO groups, demonstrating the JEO’s potent antioxidative capacity [63]. The MAP group showed superior a* value preservation versus the WOM group (*p* < 0.05) due to oxygen exclusion that prevented Mb oxidation. No significant intergroup differences were observed for the b* values (*p* > 0.05), indicating that the JEO nanoemulsion had a minimal impact on the yellowness parameter. The observed color (L* and a*) stability indicates WOMs’ potential for maintaining salmon’s visual quality during storage.

### 3.8. FAAs Content

The free amino acid content is regarded as a quality indicator for fish [75]. In this experiment, quantitative analysis was conducted on 17 types of amino acids in salmon. The total amino acid content decreased in all groups to varying degrees. The WOM-MAP group showed the smallest decline, and the untreated groups showed the greatest declines (Table 3). Free amino acids play an important role in the taste and nutritional content of fish. Glutamic acid, aspartic acid, alanine, and glycine give marine fish their distinctive flavor [75,76]. Glycine and alanine are sweet-tasting amino acids, and glutamate and aspartate are umami-tasting amino acids [77]. The glutamic acid content consistently decreased in all the groups during storage, whereas the aspartic acid, alanine, and glycine levels showed no significant increase or decrease. The WOM-MAP group had a significantly reduced decline compared to the other three groups, likely due to the WOM-MAP’s antibacterial activity. Tryptophan, phenylalanine, leucine, histidine, valine, and isoleucine are classified as bitter-tasting amino acids [78]. Except for tryptophan and histidine, the levels of these amino acids increased over time, contributing to a shift in flavor: reduced sweetness and enhanced bitterness. For aquatic organisms, arginine, lysine, and leucine are the primary essential amino acids, making them key components of high-quality protein sources from aquatic products [79]. Among the 17 amino acids, lysine showed the highest concentration. The arginine and lysine contents significantly decreased during storage, indicating a decrease in the nutritional content. Additionally, a decline in tyrosine and lysine levels can lead to the release of biogenic amines through decarboxylation.

During storage, the initial changes in the levels of amino acids resulted from enzyme-mediated autolysis (e.g., decarboxylation), while the later changes were caused by microorganisms [75]. On the 8th day, the levels of two-thirds of the amino acids had declined to some extent. On the 20th day, some amino acids had increased and even exceeded their original levels, likely because microorganisms hydrolyzed dipeptides and oligopeptides. In summary, the WOM treatment prevented the degradation of both proteins and amino acids, allowing the fish to maintain its original flavor and nutritional content.

### 3.9. K Value

During storage, ATP degrades in the sequence of ATP-ADP-AMP-IMP-HxR-Hx-uric acid [80]. The K value is used to measure the extent of ATP degradation and fish freshness [3]. IMP is the main umami compound in aquatic products, while Hx is a bitter-tasting substance [81,82]. Therefore, the degradation of IMP into Hx is induced by both autolytic and microbial enzymes and inevitably leads to an undesirable flavor [82]. Marine fish with a K value below 15% are generally considered high quality, while those with a K value exceeding 60% are no longer suitable for consumption [62]. The initial K value was 37% (Figure 5D), which was relatively higher than the normal level and indicated that ATP degradation had begun. Souza et al. [83] reported that the initial K value of the same species was 10%; this difference could be due to various factors (e.g., storage pressure and temperature after capture) that affected the K values. The K values increased continuously during storage, with the CK and WO groups exceeding the threshold on the 8th day, while the WOM and WOM-MAP groups reached 60% on the 12th day and 16th day, significantly extending the preservation period. During storage, the ATP, ADP, AMP, and IMP contents decreased, while Hx and HxR accumulated [66]. The degradation of ATP and IMP is due to microbial enzyme activity and autolytic processes [84]. The lower K values in the WOM and WOM-MAP groups can be attributed to the presence of the essential oil, which inhibited microbial growth and prevented enzyme secretion, thereby slowing the ATP **→** HxR conversion and reducing Hx and HxR accumulation [66,81]. Furthermore, the WOM-MAP group showed significantly slower degradation than the WOM group due to the MAP. Bacteria proliferate more rapidly in the presence of oxygen, whereas a CO_2_ and N_2_ environment suppresses the growth and activity of spoilage bacteria [3,27]. WOM and WOM-MAP largely slowed down the degradation of ATP, allowing the salmon to maintain its freshness and extending the shelf life by at least 4 days during storage.

### 3.10. Myofibrillar Proteins (MPs)

#### 3.10.1. MP Carbonyl Group Content 

**Figure 6:**
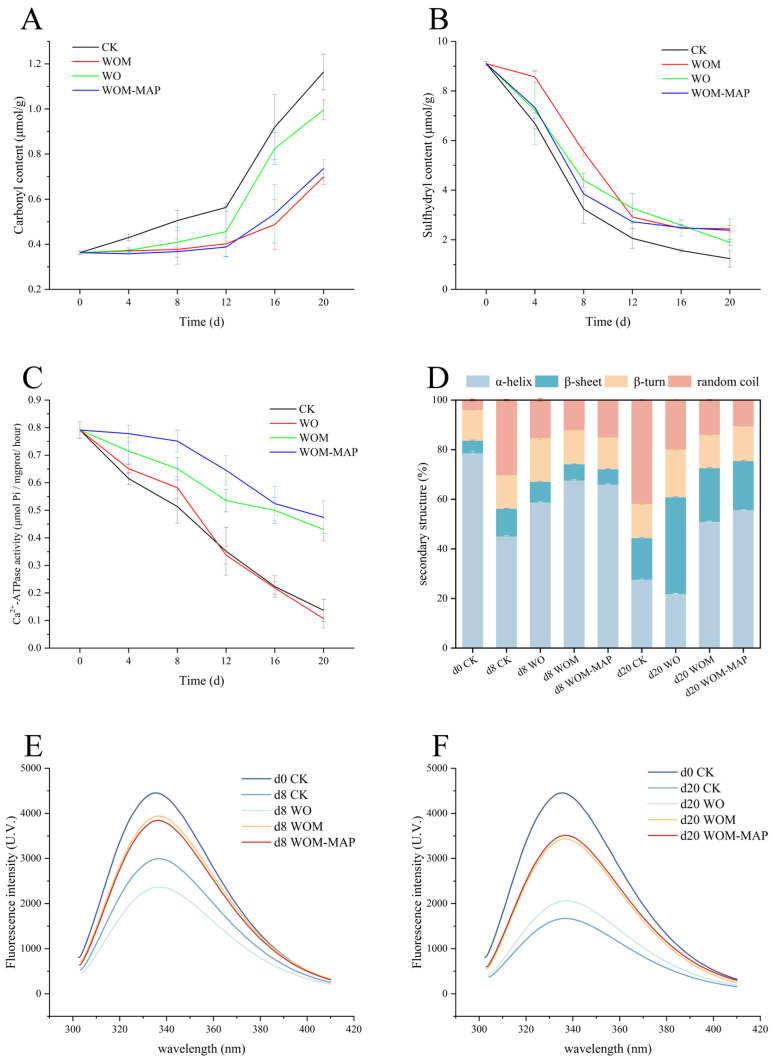
Changes in MP primary structures: carbonyl group content (**A**), sulfhydryl group content (**B**), Ca^2+^-ATPase activity (**C**); changes in MP secondary structures (**D**) and MP tertiary structures (fluorescence intensity) (**E**,**F**) using different packaging methods during storage.

During storage, proteins in fish undergo oxidation, resulting in structural and functional changes that lead to quality deterioration. Protein oxidation involves covalent modifications, backbone cleavage, and crosslinking [85]. The formation of protein carbonyl groups is commonly used to measure the extent of protein oxidation [86]. The initial carbonyl content of the fresh salmon was 0.36 μmol/g. Figure 6A shows that all the groups exhibited a similar trend of an increasing carbonyl content over time, though at different rates. The CK group had the fastest increase, while the WOM-MAP group had the slowest, followed by the WOM group. Throughout storage, the MP carbonyl group levels of the WOM group and WOM-MAP group were always below that of the CK group. The final carbonyl group content of the WOM and WOM-MAP groups remained lower than that of the CK group. On the 20th day, the final carbonyl contents in the WOM and WOM-MAP groups were 0.70 μmol/g and 0.73 μmol/g, respectively, which was significantly lower than that of the CK group (1.16 μmol/g). These results demonstrate that the JEO-loaded NE could effectively slow protein oxidation, consistent with the findings of Konfo [87]. JEO acts as an antioxidant by scavenging free radicals and ROS, likely due to its primary bioactive compounds cinnamylaldehyde and linalool [11,63]. The WOM treatment effectively slowed the formation of carbonyl groups by eliminating ROS and inhibiting the reproduction of bacteria.

#### 3.10.2. MP Sulfhydryl Group Content 

The sulfhydryl group content serves as an indicator of MP aggregation and denaturation [88]. The sulfhydryl group (-SH) of cysteine is readily oxidized by ROS, forming a disulfide bond (S-S) with another cysteine residue. S-S bond formation promotes protein cross-linking, which affects fish texture development [86,89]. Additionally, disulfide bond formation contributes to protein denaturation [90]. The initial sulfhydryl group content in the fresh salmon samples on day 0 was 9.10 μmol/g. As shown in Figure 6B, all the groups exhibited a gradual decrease in sulfhydryl group content during storage. The CK group showed the most rapid decline, dropping from 9.10 μmol/g (on day 0) to 1.56 μmol/g (on day 20). In contrast, the WOM and WOM-MAP groups demonstrated a slower reduction, reaching 2.44 and 2.389 μmol/g on the 20th day, respectively. These results confirm that the JEO-loaded NE effectively inhibited protein oxidation and disulfide bond formation, aligning with previous findings. Thus, JEO-loaded NE treatment represents a viable method for mitigating MP oxidation in stored fish.

#### 3.10.3. Ca^2+^-ATPase Activity of MPs

Ca^2+^-ATPase activity serves as an indicator of myofibrillar integrity, reflecting the functional state of myosin globular heads [91]. During storage, the Ca^2+^-ATPase activity declined in all the treatment groups (Figure 6C). The initial Ca^2+^-ATPase activity was 0.79 μmol Pi/mg prot/h. Notably, the WOM and WOM-MAP groups maintained higher Ca^2+^-ATPase activities than the CK and WO groups throughout the storage period. Previous studies indicated that ROS primarily regulate Ca^2+^-ATPase activity [92]. The enzyme’s active center contains a cysteine (Cys) sulfhydryl group and oxidation of this residue by ROS directly reduces Ca^2+^-ATPase activity [93]. Therefore, decreases in sulfhydryl group content and Ca^2+^-ATPase activity often occur simultaneously [94]. Additionally, protein structural degradation also contributes to declining activity. These results demonstrate that the JEO-loaded NE’s antioxidant properties effectively mitigated Ca^2+^-ATPase inactivation, corroborating earlier findings. Furthermore, Ca^2+^-ATPase activity correlates with secondary structural changes, particularly changes to the β-sheet/α-helix ratio [86]. As a result, the WOM helped maintained the salmon’s myofibrillar integrity to a great extent.

#### 3.10.4. MP Secondary Structures

The secondary structure of MPs includes α-helices, β-sheets, β-turns, and random coil. The content of each type of structure can be measured using circular dichroism spectroscopy. Previous studies have shown that α-helices are the dominant configuration in the rod portion of MPs, accounting for 95% [95]. High α-helix and β-sheet contents typically indicate protein regularity, while high β-turn and random coil contents reflect flexibility and structural relaxation [96,97]. The initial MP secondary structure in the fresh samples consisted of 78.67% α-helices, 5.02% β-sheets, 12.44% β-turns, and 3.87% random coils (Figure 6D). With extended storage time, the α-helix content significantly decreased, while the β-turn and random coil contents increased. Additionally, the β-sheet content remained relatively stable in the early storage stage but generally increased in later stages. The reduction in the α-helix content was due to MP denaturation. The stability of α-helices depends on hydrogen bonds within the peptide backbone [53,98]. Microbial degradation of the protein disrupts these hydrogen bonds, triggering a structural transition from α-helices to other configurations (β-turns and random coils) [99]. Compared to α-helices, β-sheets have fewer hydrogen bonds, making them more flexible and less capable of maintaining structural integrity, ultimately affecting the texture (e.g., hardness and springiness) [100]. On the 20th day, the treated groups exhibited significantly higher α-helix contents than the untreated group, indicating that the JEO-loaded NE effectively preserved the secondary structure due to its antibacterial activity. In a conclusion, the WOM significantly stabilized the secondary structure.

#### 3.10.5. MP Tertiary Structures

The intensity of the intrinsic fluorescence, primarily emitted by tryptophan and tyrosine, serves as a key indicator of protein tertiary structures [95,101]. Endogenous tryptophan is particularly useful for monitoring tertiary structure changes due to its high fluorescence intensity and sensitivity to microenvironmental alterations [97]. When myofibrillar proteins (MPs) undergo oxidation or denaturation, their structure becomes more expanded, causing the tryptophan residues in the protein core to become exposed [102]. This leads to MP aggregation and a decrease in intrinsic fluorescence intensity [103]. All the experimental groups exhibited fluorescence peaks near 330 nm, which is a typical fluorescence spectrum for endogenous tryptophan (Figure 6E,F). In the CK group, the fluorescence intensity sharply decreased during storage, indicating that the tryptophan was exposed to a more hydrophilic microenvironment and the protein was denatured [104]. However, the treated groups consistently maintained higher fluorescence levels compared to both the CK and WO groups. These results demonstrate that the JEO-loaded NE effectively preserved the tertiary structure due to its antioxidative and antibacterial properties, which slowed tryptophan exposure. Additionally, the MAP contributed to maintaining MP structural integrity through oxygen and CO_2_ isolation. This packaging method also partially inhibited microbially mediated protein degradation [97]. The WOM treatment effectively slowed down the degradation of the tertiary structure due to denaturation.

## 4. Conclusions

The application of a JEO-loaded-NE to Atlantic salmon preserved using cold storage effectively extended its shelf life while maintaining the original flavor. The WOM treatment inhibited microbial reproduction (extending the shelf life to at least 16 days compared to CK’s 8 days) while preventing significant changes in the pH and TVB-N values. The MP structural analysis revealed that the WOM treatment effectively slowed the oxidation and denaturation of carbonyl and sulfhydryl groups while maintaining both the secondary and tertiary structures. Based on the TPA, FAA, and K value results, the WOM treatment was found to be able to maintain the fish’s original flavor characteristics and minimize nutrient losses, which was primarily attributed to the antibacterial and antioxidative properties of the JEO-loaded NE. The research convincingly demonstrated that the JEO-loaded NE is an effective natural preservative, extending the shelf life of salmon and helping it to maintain its original flavor during refrigerated preservation. Additionally, this study proved that the overall effects of the WOM-MAP were superior to those of the WOM, particularly in inhibiting microbial spoilage, demonstrating the tremendous potential of the combination of a JEO-loaded NE and other preservation methods to enhance the effects of preservation. Future research should focus on elucidating the antibacterial and antioxidative mechanisms of JEO-loaded NEs and compare their preservative abilities with those of current preservation methods. 

## Figures and Tables

**Table 1:** Different packaging methods for the four groups. Control groups: CK (blank) and WO; experimental groups: WOM and WOM-MAP.

Group	Packaging Material	Modified Atmosphere Packaging
Control Check (CK)	-	-
WO	WP-OCS complex	-
WOM	JEO-loaded nanoemulsion	-
WOM-MAP	JEO-loaded nanoemulsion	N_2_: 60%; CO_2_: 40%

**Table 2:** Diameter of the inhibition zone and minimum inhibitory concentration (MIC) of JEO-loaded nanoemulsion against three different bacterial strains.

	Inhibition Zone Diameter (mm)	MIC (μL/mL)
*Staphylococcus aureus*	14.0 ± 1.0	5
*Shewanella putrefaciens*	13.5 ± 0.5	10
*Pseudomonas fluorescens*	21.5 ± 2.0	10

**Table 3:** Changes in free amino acid (FAA) content (mg/100 g) using different packaging methods during storage.

Group	Asp	Thr	Ser	Glu	Gly	Ala
d0CK	4.25 ± 0.18 ^d^	35.14 ± 1.32 ^a^	25.94 ± 0.91 ^e^	43.35 ± 1.55 ^a^	67.24 ± 1.91 ^ab^	143.44 ± 4.35 ^d^
d8CK	2.26 ± 0.05 ^f^	26.76 ± 0.58 ^f^	29.24 ± 0.94 ^c^	3.08 ± 0.09 ^f^	70.11 ± 1.65 ^a^	173.55 ± 4.26 ^a^
d8WO	0.93 ± 0.03 ^g^	29.18 ± 1.1 ^d^	13.62 ± 0.65 ^g^	7.8 ± 0.33 ^e^	67.28 ± 2.29 ^ab^	162.77 ± 6 ^b^
d8WOM	5.7 ± 0.3 ^c^	26.11 ± 1.15 ^f^	21.67 ± 1.37 ^f^	23.07 ± 1.27 ^c^	58.77 ± 2.77 ^d^	151.15 ± 7.45 ^c^
d8WOM-MAP	10.23 ± 0.07 ^b^	33.19 ± 0.18 ^b^	33.22 ± 0.16 ^b^	36 ± 0.22 ^b^	52.13 ± 0.12 ^e^	133.87 ± 0.21 ^e^
d20CK	3.55 ± 0.1 ^e^	27.53 ± 0.77 ^ef^	27.48 ± 0.85 ^d^	2.69 ± 0.17 ^f^	66.81 ± 1.82 ^b^	166.3 ± 4.63 ^b^
d20WO	2.35 ± 0.07 ^f^	28.31 ± 0.36 ^de^	22.54 ± 0.37 ^f^	2.31 ± 0.03 ^f^	66.65 ± 0.2 ^b^	160.13 ± 0.45 ^b^
d20WOM	4.51 ± 0.18 ^d^	26.13 ± 0.47 ^f^	33 ± 0.26 ^b^	2.93 ± 0.04 ^f^	63.36 ± 0.4 ^c^	162.05 ± 1.16 ^b^
d20WOM-MAP	13.15 ± 0.19 ^a^	31.73 ± 0.58 ^c^	44.99 ± 0.49 ^a^	12.26 ± 0.35 ^d^	56.93 ± 0.95 ^d^	151.99 ± 2.05 ^c^
Group	Cyst	Val	Met	Ile	Leu	Tyr
d0CK	11.7 ± 0.18 ^a^	24.44 ± 0.77 ^f^	7.3 ± 0.28 ^f^	9.5 ± 0.35 ^e^	24.66 ± 0.87 ^g^	26.56 ± 16.21 ^a^
d8CK	11.03 ± 0.19 ^b^	35.36 ± 0.91 ^c^	10 ± 0.26 ^d^	11.96 ± 0.32 ^d^	39.4 ± 1.15 ^d^	24.88 ± 0.54 ^a^
d8WO	10.97 ± 0.41 ^b^	28.27 ± 1.17 ^e^	7.33 ± 0.33 ^f^	7.74 ± 0.29 ^f^	26.11 ± 0.96 ^fg^	0.65 ± 0.16 ^b^
d8WOM	9.92 ± 0.53 ^c^	27.05 ± 1.41 ^e^	6.99 ± 0.37 ^f^	7.48 ± 0.43 ^f^	27.01 ± 1.37 ^f^	20.13 ± 1.12 ^a^
d8WOM-MAP	8.13 ± 0.08 ^e^	31.15 ± 0.05 ^d^	9.47 ± 0.1 ^e^	11.74 ± 0.08 ^d^	35.4 ± 0.15 ^e^	21.05 ± 0.11 ^a^
d20CK	8.19 ± 0.28 ^e^	43.08 ± 1.29 ^a^	16.58 ± 0.47 ^bc^	16.37 ± 0.46 ^a^	46.03 ± 1.40 ^b^	1.98 ± 0.1 ^b^
d20WO	7.73 ± 0.09 ^e^	42.99 ± 0.07 ^a^	17.09 ± 0.04 ^b^	15.54 ± 0.08 ^b^	40.15 ± 0.04 ^d^	18.35 ± 0.08 ^a^
d20WOM	9.14 ± 0.25 ^d^	40.02 ± 0.36 ^b^	16.28 ± 0.17 ^c^	14.1 ± 0.17 ^c^	42.04 ± 0.39 ^c^	17.86 ± 0.16 ^a^
d20WOM-MAP	7.77 ± 0.26 ^e^	41.93 ± 0.68 ^a^	19.16 ± 0.39 ^a^	16.54 ± 0.26 ^a^	48.42 ± 0.93 ^a^	19.73 ± 0.08 ^a^
Group	Phe	Lys	His	Arg	Pro	Total
d0CK	16.94 ± 0.62 ^e^	478.78 ± 20.41 ^a^	138.13 ± 5.49 ^a^	7.60 ± 0.30 ^a^	5.91 ± 0.52 ^f^	1288.24 ± 47.12 ^a^
d8CK	31.59 ± 0.35 ^a^	456.61 ± 12.69 ^a^	43.52 ± 1.34 ^d^	0.68 ± 0.06 ^de^	9.51 ± 0.69 ^d^	1038.16 ± 19.36 ^b^
d8WO	20.63 ± 0.78 ^d^	380.6 ± 18.42 ^cd^	10.05 ± 0.07 ^g^	0.46 ± 0.02 ^f^	8.11 ± 0.77 ^e^	832.82 ± 36.65 ^f^
d8WOM	25.96 ± 1.71 ^c^	418.53 ± 25.88 ^b^	51.52 ± 3.04 ^c^	0.77 ± 0.07 ^d^	6.34 ± 0.95 ^f^	921.5 ± 50.57 ^cde^
d8WOM-MAP	28.08 ± 0.14 ^b^	370.52 ± 1.42 ^cd^	64.81 ± 0.13 ^b^	1.14 ± 0.03 ^c^	7.94 ± 0.45 ^e^	931.39 ± 1.03 ^cd^
d20CK	25.34 ± 0.68 ^c^	393.13 ± 11.85 ^c^	18.55 ± 0.59 ^f^	0.58 ± 0.01 ^def^	13.98 ± 0.29 ^b^	938.19 ± 26.72 ^cd^
d20WO	16.91 ± 0.05 ^e^	356.3 ± 0.99 ^d^	1.54 ± 0.03 ^h^	0.51 ± 0.01 ^ef^	15.38 ± 0.04 ^a^	870.31 ± 0.93 ^ef^
d20WOM	26.06 ± 0.36 ^c^	373.61 ± 4.62 ^cd^	6.71 ± 0.16 ^g^	0.64 ± 0.02 ^def^	11.24 ± 0.06 ^c^	902.97 ± 8.78 ^de^
d20WOM-MAP	30.79 ± 0.34 ^a^	363.06 ± 4.18 ^d^	33.9 ± 0.75 ^e^	1.63 ± 0.02 ^b^	11.33 ± 1.03 ^c^	965.05 ± 14.72 ^c^

Values in the same column with different lowercase letters are significantly different (*p* < 0.05).

## Data Availability

The original contributions presented in this study are included in the article. Further inquiries can be directed to the corresponding author.

## Abbreviations

The following abbreviations are used in this manuscript:

JEO	jasmine essential oil
OCS	oxidized corn starch
WP	whey protein
MAP	modified atmosphere packaging
CK	control check
WO	whey protein–oxidized corn starch mixture
NE	nanoemulsion
QIM	quality index technique
PDI	polydispersity index
MIC	minimum inhibitory concentration
TPA	texture profile analysis
TVB-N	total volatile basic nitrogen
TVC	total viable count
PCA	perchloric acid
FAAs	free amino acids
Hx	hypoxanthine
HxR	hypoxanthine ribonucleoside
IMP	inosine monophosphate
AMP	adenosine monophosphate
ADP	adenosine diphosphate
ATP	adenosine triphosphate
HPLC	high-performance liquid chromatography
MPs	myofibrillar proteins
